# Short Term Prognosis of Renal Artery Stenosis Secondary to Acute Type B Aortic Dissection With TEVAR

**DOI:** 10.3389/fcvm.2021.658952

**Published:** 2021-04-23

**Authors:** Lei Li, Maozhou Wang, Jinzhang Li, Xinliang Guan, Pu Xin, Xiaolong Wang, Yuyong Liu, Haiyang Li, Wenjian Jiang, Ming Gong, Hongjia Zhang

**Affiliations:** ^1^Department of Cardiac Surgery, Beijing Anzhen Hospital, Capital Medical University, Beijing, China; ^2^Beijing Aortic Disease Center, Cardiovascular Surgery Center, Beijing, China; ^3^Beijing Lab for Cardiovascular Precision Medicine, Beijing, China; ^4^Department of Medical Imaging, Beijing Anzhen Hospital, Capital Medical University, Beijing, China

**Keywords:** renal artery stenosis, acute type B aortic dissection, acute kidney injury, hypertension, early prognosis

## Abstract

**Objective:** To determine the effect of renal artery stenosis (RAS) resulting from acute type B aortic dissection (ATBAD) with thoracic endovascular aortic repair (TEVAR) on early prognosis in patients with ATBAD.

**Methods:** A total of 129 ATBAD patients in the National Acute Aortic Syndrome Database (AASCN) who underwent TEVAR between 2019 and 2020 were enrolled in our study. Patients were divided into two groups: the RAS group and the non-RAS group.

**Results:** There were 21 RAS patients (16.3%) and 108 non-RAS patients (83.7%) in our cohort. No patient in our cohort died during the 1-month follow-up. There was no significant difference in preoperative creatinine clearance rate (CCr) between the two groups (90.6 ± 46.1 μmol/L in the RAS group vs. 78.7 ± 39.2 μmol/L in the non-RAS group, *P* = 0.303) but the RAS group had a significantly lower estimated glomerular filtration rate (eGFR) than the non-RAS group (83.3 ± 25.0 vs. 101.9 ± 26.9 ml/min, respectively; *P* = 0.028).One month after TEVAR, CCr was significantly higher (99.0 ± 68.1 vs. 78.5 ± 25.8 ml/min, *P* = 0.043) and eGFR (81.7 ± 23.8 vs. 96.0 ± 20.0 ml/min, *P* = 0.017) was significantly lower in the RAS group than in the non-RAS group.

**Conclusions:** In ATBAD, RAS could result in acute kidney injury (AKI) in the early stage after TEVAR. The RAS group had a high incidence of hypertension. These results suggest that patients with RAS may need further treatment.

## Introduction

Acute type B aortic dissection (ATBAD) refers to dissection involving the distal left subclavian artery (1, 2). Renal artery involvement (RAI) is one of the common complications of ATBAD, with an incidence rate of 45–48% (3, 4). Some researchers found that RAI did not affect the perioperative renal function of patients with ATBAD. Based on the results of their study, they concluded that the RAI caused by ATBAD can be treated conservatively (3, 5). Previous studies on RAI in patients with ATBAD have demonstrated little detailed classification of renal artery injury.

Renal artery stenosis (RAS) has been defined as a reduction of more than 60% in luminal diameter (6) that may lead to refractory hypertension and a progressive decline in renal function (3, 7). Non-dissection-related RAS is mostly caused by hemodynamic compression and atherosclerosis, which may be related to atherosclerotic inflammation (8). Moreover, RAS induced by ATBAD is mostly caused by hematoma compression or renal artery dissection. However, it is not clear whether RAS caused by ATBAD will affect prognosis after TEVAR (endovascular repair of type B aortic dissection). We found that few of these studies clearly distinguished RAS from RAI. Postoperative acute kidney injury (AKI) and refractory hypertension are common complications and severely impact the prognosis after TEVAR (9, 10). Renal artery stenosis but not RAI could be one of the reasons for these complications. Therefore, verifying the impact of RAS on the prognosis of these patients is the key to improving the quality of life of ATBAD patients.

The National Key Research and Development Project database is based on the AASCN (acute aortic syndrome cooperation network) database and is supported by the Ministry of Science and Technology of the People's Republic of China, the Ministry of Education of the People's Republic of China and Beijing Municipal Commission of Science and Technology in 2018. At present, more than 2,500 patients with aortic syndrome and more than 11,000 specimens have been collected. The scale of the database ranks in the forefront in China, and the aortic dissection data can cover most people with aortic dissection in China. This study aims to focus on the effects of ATBAD-induced RAS on early renal function and hypertension after TEVAR.

## Methods

### Patients

The AASCN database contains data from patients who suffered from acute aortic syndrome at 10 heart centers in China. We used RAS with ATBAD as an exposure factor to assemble a study cohort from the AASCN and eliminated patients without ATBAD or with a lack of follow-up. All ATBAD patients who received TEVAR were enrolled in our study. Patients with conservative treatment (*n* = 79), open surgery (*n* = 3), preoperative kidney disease [Including preoperative polycystic kidney (*n* = 4), renal calculi (*n* = 6), renal atherosclerotic stenosis (*n* = 9), and unilateral kidney (*n* = 2)] were eliminated from our study. Ultimately, 129 ATBAD patients in the AASCN database who underwent TEVAR were enrolled in our study. We observed patients from their arrival at the hospital until 1-month after TEVAR. We divided these patients into the RAS group and the non-RAS group. This study was mainly led by Anzhen Hospital, Beijing, China, and approved by the hospital's Ethics Committee in April 2018 (No. 2018004). The Chinese Clinical Trial Registry (ChiCTR) number is ChiCTR1900022637. The procedures were in accordance with the ethical standards of the responsible committee on human experimentation.

### Definitions and End-Point

**Figure 1:**
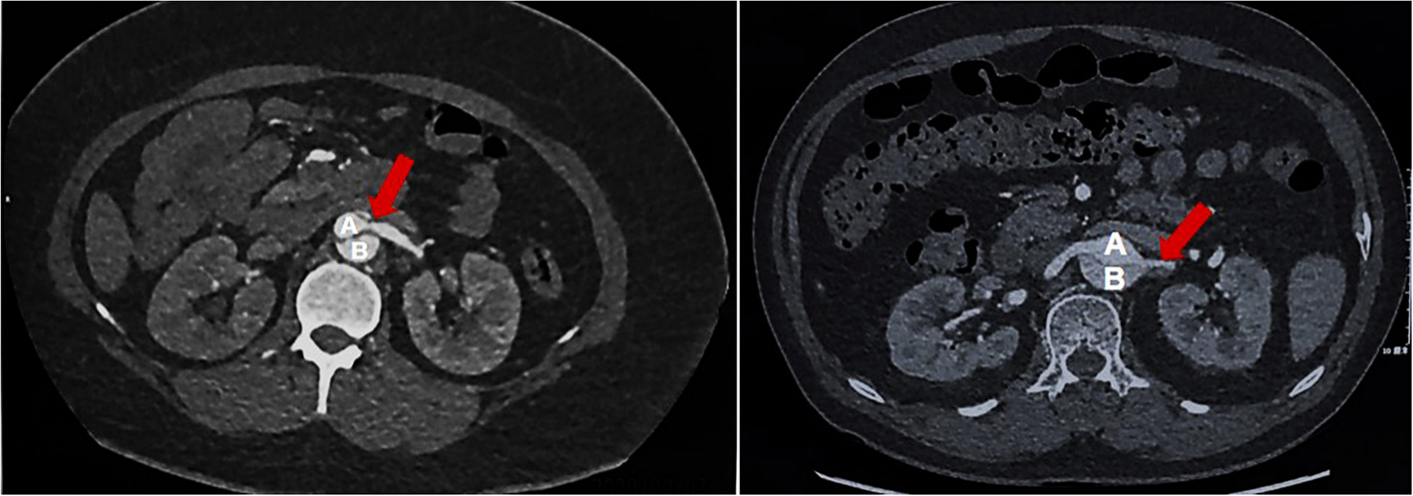
RAS group: Renal artery stenosis group, a reduction of more than 60% in the effective renal artery lumen diameter on one or both sides; **(A)** True lumen of aortic; **(B)** False lumen of aortic; Red Arrow: Stenosis of renal artery.

**Figure 2:**
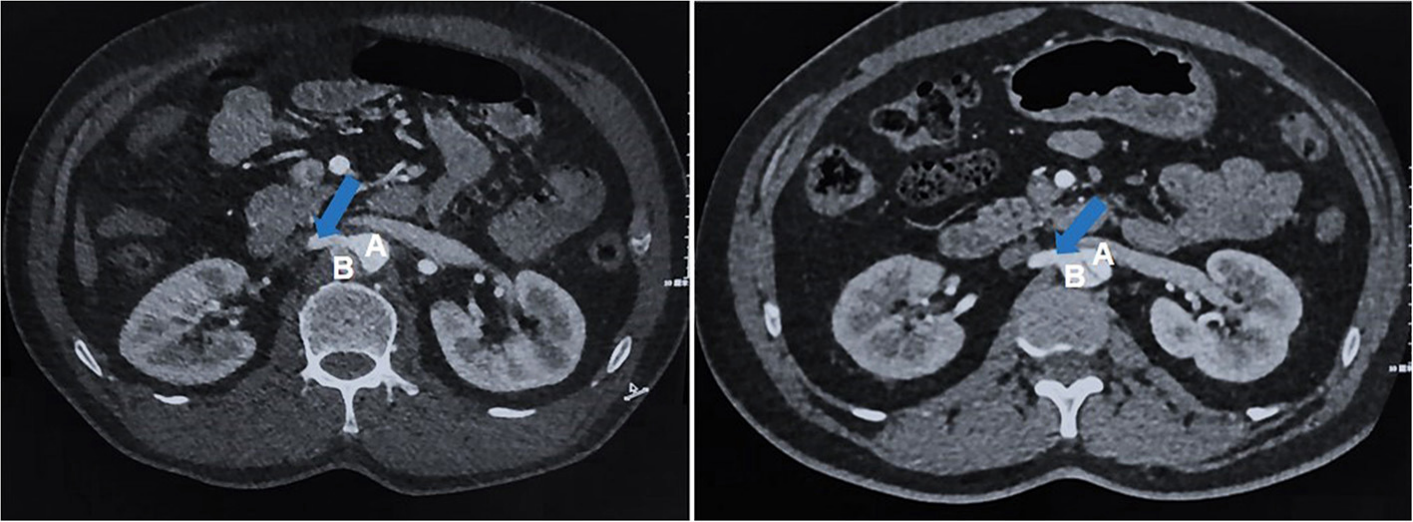
Non-RAS group: No renal artery stenosis group, both renal artery lumen effective diameters maintained at or above 40%, regardless of dissection involvement; **(A)** True lumen of aortic; **(B)** False lumen of aortic; Blue Arrow: Without stenosis of renal artery.

The diagnoses of RAS and non-RAS were based on preoperative aortic computed tomography. Aortic computed tomography was observed and measured by senior imaging doctors who are good at the diagnosis of vascular diseases (more than 200 cases of aortic related diseases are diagnosed each year). Renal artery stenosis was defined as a reduction of more than 60% in the effective renal artery lumen diameter on one or both sides (Figure 1). Non-RAS was defined as both renal artery lumen effective diameters maintained at or above 40%, regardless of dissection involvement. Renal artery involvement flow limiting dynamic hemodynamic compression, non-flow limiting static dissection, flow limiting static dissection, or false lumen blood-supply according to previous study (11) (Figure 2). Therefore, some patients with RAI were included in the non-RAS group. Although the renal artery was affected (false lumen blood supply, intima formation), the effective lumen diameter of the renal artery remained within the normal range. Acute kidney injury was defined as a 50% increase in creatinine within 7 days, an increase in creatinine by 26 μmol/L within 2 days or oliguria according to KDIGO baseline. The estimated glomerular filtration rate (eGFR) was estimated by the Cockcroft–Gault formula ((140 – age) × body weight)/(72 × creatinine) with adjustment for sex (×0.85 for women) (12, 13). The primary end-point was AKI. The secondary outcome was hypertension [systolic blood pressure (SBP) >140 mmHg or diastolic blood pressure (DBP) >90 mmHg] (14). Thoracic endovascular aortic repair was suitable for patients with ATBAD whose proximal end is more than 2 cm away from the left subclavian artery. All the patients were treated with stent graft alone.

### Statistical Analysis

Continuous variables were analyzed via independent-sample *t*-tests if they obeyed a normal distribution. The Wilcoxon rank sum test was used to analyze continuous variables that did not obey the normal distribution. All continuous variables are expressed as the mean with a standard deviation (SD) or median with an interquartile range (IQR). Categorical variables are presented as frequencies with percentages and were analyzed by chi-square or Fisher's exact test, as appropriate. Two-tailed *P*-values < 0.05 indicated statistical significance. We used R Programming Language version 3.4.3 (15) for all the above analyses.

## Results

### Baseline Characteristics

All patients included in this study underwent TEVAR treatment. There was no significant difference in baseline data between the RAS population and the non-RAS population. As shown in Table 1, the age range in both groups was large. The majority of ATBAD patients treated with TEVAR were males. In the RAS group, RAI accounted for the highest proportion (61.9%). The remaining patients had RAS that was mainly secondary to hematoma compression (38.1%). In the non-RAS group, 52 patients (48.1%) suffered from RAI. The ejection fraction (EF) values of the two groups were kept in the normal range. Although the D-dimer values of the two groups were abnormal, no abnormal changes were found in other coagulation system indexes. The liver and circulatory system indicators also did not show significant changes.

**Table 1:** Baseline characteristics and multi system performance in study groups.

**Variable**	**RAS**^**a**^ **group**	**non-RAS group**	***P*-value**
	**(*n* = 21)**	**(*n* = 108)**	
General information			
Age, mean(SD)	63.9 ± 15.0	58.6 ± 13.5	0.146
Male, *n*(%)	16(80.0)	91(86.7)	0.436
BMI^b^, mean(SD) (Kg/m^2^)	27.2 ± 2.7	26.6 ± 4.2	0.617
Heart rate, mean(SD)	78.4 ± 12.0	78.8 ± 10.2	0.875
Previous history			
Smoking, *n*(%)	7(33.3)	32(29.6)	0.735
History of previous heart surgery, *n*(%)	0(0)	1(1.0)	0.652
Hypertension, *n*(%)	16(76.2)	69(63.9)	0.277
Coronary heart disease, *n*(%)	1(4.8)	6(5.6)	0.883
Diabetes, *n*(%)	1(4.8)	3(2.8)	0.631
Stroke, *n*(%)	3(14.3)	10(9.3)	0.484
Marfan's symdrome, *n*(%)	0(0.0)	1(0.9)	0.658
Renal artery condition			
Normal, *n*(%)	0(0.0)	56(51.9)	
Hematoma compression, *n*(%)	8(38.1)	0(0.0)	
RAI^c^, *n*(%)	13(61.9)	52(48.1)	
Laboratory examination			
EF^d^, mean(SD) (%)	65.6 ± 5.3	63.3 ± 5.0	0.086
Platelet, mean(SD) (10^9^/L)	205.7 ± 93.1	234.8 ± 96.5	0.217
hemoglobin, mean(SD) (g/L)	147.2 ± 15.6	149.1 ± 17.0	0.061
sensitivity troponin I(SD)(μg/L)	0.1 ± 0.3	0.1 ± 0.7	0.882
ALT^e^, mean(SD)(U/L)	28.1 ± 14.4	28.7 ± 26.8	0.921
AST^f^, mean(SD)(U/L)	23.8 ± 8.0	28.1 ± 38.6	0.618
D-dimer, median(IQR) (ng/ml)	979(1542)	844.5(1302.6)	0.370
WBC^g^, mean(SD) (10^9^/L)	11.7 ± 4.4	10.6 ± 4.8	0.331

a *RAS, Renal artery stenosis*;

b *BMI, body mass index*;

c *RAI, renal artery involvement*;

d *EF, ejection fraction*;

e *ALT, alanine aminotransferase*;

f *AST, aspertate aminotransferase*;

g *WBC, White blood cells; P < 0.05*.

### Primary Outcome

As shown in Table 2, the RAS group had significantly more patients with AKI than the non-RAS group (6/21 vs. 10/108; *P* = 0.014). The preoperative eGFR was significantly lower in the RAS group than in the non-RAS group (83.3 vs. 101.9 ml/min; *P* = 0.028). Moreover, after 1 month of follow-up, the creatinine clearance rate (CCr) was significantly higher (99.0 vs. 78.5 μmol/L group; *P* = 0.043), and eGFR was significantly lower (81.7 vs. 96.0 ml/min; *P* = 0.017) in the RAS group than in the non-RAS group. As shown in Table 3, RAS (OR: 4.977; 95% confidence interval: 1.064–23.283) and preoperative CCr (OR: 1.046; 95% confidence interval: 1.009–1.085) were independent risk factors for renal dysfunction after the 1-month follow-up.

**Table 2:** Renal function index.

**Variable**	**RAS** ^**a**^ **group**	**non-RAS group**	***P*-value**
	**(*n* = 21)**	**(*n* = 108)**	
Acute kidney injury, *n*(%)	6(28.6)	10(9.3)	0.014^*^
Preoperative Ccr^b^(SD), μmol/L	90.6(46.1)	78.7(39.2)	0.303
Preoperative eGFR^c^(SD), ml/min	83.3(25.0)	101.9(26.9)	0.028^*^
CCr after 1-month follow-up(SD), μmol/L	99.0(68.1)	78.5(25.8)	0.043^*^
eGFR after 1-month follow-up(SD), ml/min	81.7(23.8)	96.0(20.0)	0.017^*^

a *RAS, renal artery stenosis*;

b *Ccr, creatinine clearance rate*;

c *eGFR, estimated glomerular filtration rate*.

* *P < 0.05*.

**Table 3:** Variables in logistic regression model of renal dysfunction 1-month follow-up.

**Variable**	**OR**	**95%CI**	***P*-value**
RAS	4.977	1.064–28.283	0.041^*^
Preoperative Ccr	1.046	1.009–1.085	0.015^*^
Hypertension	2.325	0.372–14.541	0.367
Smoking	2.180	0.405–11.731	0.364
RAI	1.100	0.222–5.457	0.907

*RAS, renal artery stenosis; Ccr, creatinine clearance rate; RAI, renal artery involvement*.

* *P < 0.05*.

### Secondary Outcome

As shown in Table 4, preoperative DBP (*P* = 0.145) and SBP (*P* = 0.130) were not significantly different between the two groups. However, after the 1-month follow-up, SBP was significantly higher in the RAS group than in the non-RAS group (146.9 vs. 136.8 mmHg, *P* = 0.045), but DBP was not significantly different between the two groups.

**Table 4:** Blood pressure.

**Variable**	**RAS** ^**a**^**group**	**non-RAS group**	***P*-value**
	**(*n* = 21)**	**(*n* = 108)**	
Preoperative SBP^b^ ± SD^c^, mmHg	137.5 ± 16.6	134.5 ± 18.4	0.491
Preoperative DBP^d^ ± SD, mmHg	80.3 ± 14.1	76.8 ± 10.9	0.199
SBP after 1-month follow-up ± SD, mmHg	146.9 ± 18.1	136.8 ± 21.3	0.045^*^
DBP after 1-month follow-up ± SD, mmHg	79.8 ± 9.2	78.6 ± 10.8	0.635

a *RAS, renal artery stenosis*;

b *SBP, systolic blood pressure*;

c *SD, standard deviation*;

d *DBP, diastolic pressure*;

* *P < 0.05*.

## Discussion

Acute aortic dissection is an acute disease with rapid progression and high mortality (16). Since 1999, thoracic aortic stents (TEVARs) have been gradually used in patients with type B aortic dissection (17). However, there is no consensus on the changes in organ function after TEVAR, especially changes in renal function and the corresponding treatment (18). Perioperative AKI during aortic dissection has long troubled clinicians (2). The current research on renal artery status and renal function is confusing. Some studies have shown that RAI is not related to renal function (3, 5). In these studies, renal function was compared between patients with RAI and those without. Non-dissection-related RAS seriously affects the prognosis of patients and is a key factor in the risk model of prognosis (19). Previous studies on non-dissection-related RAS have mostly focused on atherosclerosis (20, 21). The main innovation of this study is ability to distinguish the dissection-related RAS population from the RAI group. We found that renal function is directly related to renal blood perfusion. Renal blood perfusion in some patients with RAI is not affected, so there is no significant reduction in renal function in this population. However, renal artery perfusion is significantly affected in the dissection-related RAS population, so it is necessary to analyze renal function after TEVAR in the dissection-related RAS population. There have been many reports about the dissection-related RAI (3, 5), but there have been no reports about dissection-related RAS.

Previous treatment of dissection-related RAI is controversial (3–5, 22). Some authors suggested that TEVAR benefited patients with dissected renal arteries in chronic aortic dissection. Therefore, some experts believe that RAI after TEVAR does not need to be treated or can be observed conservatively. However, some studies have found that the renal function of some patients treated with TEVAR is worsening (23, 24). Thoracic endovascular aortic repair closes the false lumen, which interrupts the blood supply of the corresponding renal artery and eventually leads to kidney atrophy (19). The kind of ATBAD-related renal artery injury that needs to be treated is a difficult problem for clinicians. Therefore, it is necessary to further classify renal artery injury rather than simply identify it as involved or not. Zhou and colleagues suggested that RAI patients with a degree of renal malperfusion >27% need active imaging follow-up and aggressive endovascular intervention in ATBAD (25). In this study, we defined RAS as a decrease in the effective diameter of one or both renal arteries of more than 60% (6) (Figure 1). Renal artery stenosis is mainly divided into flow limiting dynamic hemodynamic compression (B1), non-flow limiting static section (B2), and flow limiting static section (B3) (11). The left of Figure 1 is B1, and the right of Figure 1 belongs to B2 or B3. This classification can more intuitively determine the degree of RAI. As we have fewer cases, there is no further study on different types. Non-dissection-related RAS can be caused by atherosclerosis and fibromuscular lesions. It is a main risk factor for cardiovascular and renal complications, which may be related to inflammatory factors produced after renal stenosis (26). For non-dissection-related RAS, artery stenting is most beneficial for patients (27). However, RAS caused by ATBAD was rarely mentioned in the past. In our study, we divided ATBAD patients into the RAS group and the non-RAS group and compared their performance before and 1 month after TEVAR.

The variation in age in the two groups was large, which was related to the wide range of onset ages of aortic dissection in China (28). The majority of Chinese ATBAD patients in both groups who underwent TEVAR treatment were men, which was consistent with the current results of male dominated ATBAD epidemiological statistics (29). Most of the patients in the RAS group were overweight compared with those in the non-RAS group, but there was no significant difference between the two groups. The proportion of hypertension in both groups was more than half. We defined RAI as when the kidney supplies blood through the false lumen or when the intima is visible at the renal artery or the opening of the renal artery (3, 5) (Figure 2). In the RAS group, the percentage of RAI was 61.9%. In this part of the RAI population, the effective diameter of the renal artery lumen on one or both sides was reduced by more than 60%. In other RAS patients, the effective diameter of the renal artery lumen was reduced by more than 60% due to hematoma compression. There was no significant difference in baseline data between the RAS group and the non-RAS group.

Primary outcome analysis showed that there was no significant difference in creatinine between the RAS group and the non-RAS group before TEVAR, but the EGFR changed differently. We propose that renal perfusion in the RAS group was abnormal before TEVAR. Previous studies have also suggested that the change in eGFR is usually earlier than that in CCr (30, 31). One month after TEVAR, we found that there were significant differences in CCr and eGFR between the RAS group and the non-RAS group. In addition, RAS had a significant effect on short-term renal function in ATBAD (Table 3). Multivariate logistic regression analysis showed that both RAS and preoperative CCr were risk factors for AKI. Although there was no significant difference in preoperative CCr between the two groups, it became a risk factor for AKI after RAS was included. This finding is also consistent with our previous test results. Hematoma compression and RAI were the most common reasons or RAS in ATBAD (Table 1). For these patients, renal artery conventional angiography is necessary to clear the hemodynamics of the involved renal artery. Renal artery interventions (renal artery stenting) may also be needed in patients with RAS. In general, early diagnosis of RAS can promote early management and improve the prognosis of renal function in such patients.

Hypertension was the secondary outcome of our study, and previous studies have shown that hypertension adverse is detrimental to the postoperative recovery of ATBAD patients (32). In our study, the mean preoperative blood pressure in the RAS group was higher than that in the non-RAS group, but SBP and DBP were not significantly different between the two groups. After the 1-month follow-up, SBP was significantly higher in the RAS group than in the non-RAS group. Previous studies have shown that non-dissection-related RAS could cause hypertension by activating the renin angiotensin system (33). This finding indicates whether patients with dissection-related RAS also have symptoms of refractory hypertension for this reason. In the non-dissection-related RAS population, renin-angiotensin inhibitors are beneficial for decreasing blood pressure (34). There has been no systematic report on whether the targeted use of renin angiotensin inhibitors can effectively control the symptoms of refractory hypertension in patients with dissection related RAS. Our results provide a theoretical basis for the treatment of refractory hypertension resulting from RAS in ATBAD. We will continue to follow up these ATBAD patients with RAS for a long time to determine the effect of renin angiotensin inhibitors on blood pressure control.

## Limitation

There are some limitations in our study. In this study, indicators such as creatinine and GFR were comprehensive indicators rather than accurate values of unilateral kidneys. Therefore, more accurate recommendations should be combined with tests for the accurate detection of unilateral renal function. The long-term follow-up (>1 month) of the subjects is still in progress, and the relevant data are not included in this study. Therefore, the long-term treatment recommendations for patients still need to be further analyzed combined with long-term follow-up data. Besides, our study has selection bias: only TEVAR patients has been enrolled in our study and lacked postoperative CTA data which did not elucidate the morphological changes of the kidney.

## Conclusion

In conclusion, ATBAD-related renal artery injury cannot be simply divided into an RAI group and a non-RAI group. Previous studies based on this grouping approach have concluded that the RAI group should be treated conservatively. In this study, renal artery injury was divided into a RAS group and a non-RAS group based on the effective perfusion lumen diameter of the renal artery. The change in eGFR occurred in the RAS group before the operation, and significant AKI occurred after the operation compared with the non-RAS group. At the same time, we found that the proportion of hypertension in the RAS group was significantly higher than that in the non-RAS group. The higher probability is related to the activation of the renin-angiotensin system induced by the change in lumen diameter. It also provides a theoretical basis for the application of renin angiotensin inhibitors in the treatment of refractory hypertension resulting from RAS in ATBAD.

## Data Availability Statement

The raw data supporting the conclusions of this article will be made available by the authors, without undue reservation.

## Ethics Statement

This study was mainly led by Anzhen Hospital, Beijing, China, and approved by the hospital's Ethics Committee in April 2018 (No. 2018004). The patients/participants provided their written informed consent to participate in this study.

## Author Contributions

All authors listed have made a substantial, direct, and intellectual contribution to the work and approved it for publication.

## Conflict of Interest

The authors declare that the research was conducted in the absence of any commercial or financial relationships that could be construed as a potential conflict of interest.
